# A cross-sectional study of high-risk human papillomavirus clustering and cervical outcomes in HIV-infected women in Rio de Janeiro, Brazil

**DOI:** 10.1186/s12885-015-1486-4

**Published:** 2015-06-23

**Authors:** Jessica L. Castilho, José Eduardo Levi, Paula M. Luz, Mary Catherine Cambou, Tazio Vanni, Angela de Andrade, Mônica Derrico, Valdiléa G. Veloso, Beatriz Grinsztejn, Ruth K. Friedman

**Affiliations:** 1 Division of Infectious Diseases, Vanderbilt University School of Medicine, Nashville, USA; 2 Virology Lab, Instituto de Medicina Tropical da Universidade de São Paulo, São Paulo, Brazil; 3 Instituto Nacional de Infectologia Evandro Chagas, Fundação Oswaldo Cruz, Rio de Janeiro, Brazil; 4 Division of Infectious Diseases and Program in Global Health, David Geffen School of Medicine at UCLA, Los Angeles, USA; 5 Departamento de Ciência e Tecnologia, Ministério da Saúde, Brasília, Brazil

**Keywords:** HPV, Women, HIV, Cervical cancer, Epidemiology

## Abstract

**Background:**

In Brazil, the rate of cervical cancer remains high despite the availability of screening programs. With ongoing vaccine development and implementation, information on the prevalence of specific HPV types is needed, particularly among high-risk populations, such as HIV-infected women.

**Methods:**

We performed a study of HIV-infected women in Rio de Janeiro, Brazil, who underwent cervical HPV genotype testing between 2005-2013. We examined the prevalence of high-risk HPV types and the patterns of high-risk HPV type clustering. Using logarithmic binomial regression, we estimated the risk of abnormal cytology by HPV genotype result.

**Results:**

Of the 562 women included, 498 (89 %) had at least one HPV type detected. 364 women (65 %) had at least one high-risk HPV type detected and 181 (32 %) had more than one high-risk type detected. HPV 58 was the most frequent HPV type detected overall (prevalence 19.8 % [95 % confidence interval 16.4–23.1]), followed by HPV 53 (prevalence 15.5 % [12.5–18.5]) and HPV 16 (prevalence 13 % [10.2–15.8]). Women infected with more than one high-risk HPV type were younger, had lower CD4+ lymphocyte counts, and were more likely to be infected with HPV 16 or 18. In adjusted analyses, presence of more than one high-risk HPV type was associated with a two-fold increased risk of abnormal cytology after adjusting for presence of individual high-risk type, age, and CD4+ lymphocyte count (adjusted prevalence ratios 1.88–2.07, all *p* <0.001). No single high-risk HPV type was statistically associated with abnormal cytology after adjusting for the presence of more than one high-risk HPV type.

**Conclusions:**

In the largest study of cervical HPV genotypes among HIV-infected women in Latin America, infection by high-risk HPV types other than 16 or 18 and infection by more than one high-risk HPV types were common. Infection by more than one high-risk type was more strongly associated with abnormal cervical cytology than any individual high-risk HPV type, highlighting the need for multi-valent HPV vaccines.

## Background

Latin America has one of the highest incidence and mortality rates of cervical cancer in the world [1]. As in other regions, in Latin America, high-risk human papillomavirus (HPV) type 16 has been strongly associated with risk of high-grade cervical dysplasia and cervical cancer [2]. In Brazil, high-risk HPV types have been observed to be highly prevalent in women with both normal and abnormal cervical cytology [3, 4].

For women with HIV infection, the risks and consequences of HPV infection are even greater. Women with HIV infection have higher prevalence of high-risk HPV types, are more likely to be infected with more than one HPV type, and are at increased risk of cervical dysplasia and cervical cancer [5–11]. HIV-infected women are more likely to have abnormal cervical cytology and dysplasia associated with high-risk HPV types other than 16 or 18 [12–14]. Among HIV-infected women in Latin America, prevalence of cervical HPV infection in women with normal cervical cytology has been estimated at 57 %, similar to the prevalence observed in HIV-infected women in Africa (57 %) and higher than that observed in HIV-infected women in Asia (31 %), Europe (32 %), or North America (31 %) [13]. A recent large study of pregnant HIV-infected women in Rio de Janeiro, Brazil, noted an overall cervical HPV prevalence of 84 %, 80 % of whom were infected by high-risk types [15]. While some studies have shown a protective effect, combination antiretroviral therapy (cART) has not consistently been observed to affect rates of cervical intraepithelial neoplasia and only modestly improves HPV infection clearance [7, 16–21].

Given their increased risk, HIV-infected women are recommended to receive frequent screening for cervical dysplasia [22]. Vaccines targeting common high-risk HPV types are effective in reducing risk of cervical dysplasia and are immunogenic in HIV-infected women [23, 24]. However, these vaccines are not yet widely available in many places in the world, particularly in low- and middle-income countries [1, 25]. As next-generation HPV vaccines are developed, knowledge of high-risk HPV epidemiology, particularly among high-risk groups such as HIV-infected women, is of critical importance.

To address this need, we performed a prevalence study of cervical HPV infection among a cohort of HIV-infected women in Rio de Janeiro, Brazil. In this study, we describe the prevalence of HPV types, patterns of clustering, and patient clinical and demographic factors associated with high-risk HPV cervical infection and abnormal cytological outcomes.

## Methods

To examine the epidemiology of type-specific HPV infections, we performed an analysis of all HIV-infected women followed at the Women’s Cohort of the Instituto Nacional de Infectologia Evandro Chagas (INI), Fundção Oswaldo Cruz, who underwent HPV genotype testing by line assay at cohort entry. This study was approved by institutional ethics review boards of INI and Vanderbilt University.

The Women’s HIV Cohort at INI is an observational study of the natural history of HIV infection in women that began in 1996. After recruitment from the general HIV clinic and signing informed consent, women complete surveys on sexual and gynecologic history and undergo routine cytology-based cervical cancer screening by Papanicolaou (Pap) tests and hybrid capture detection of oncogenic HPV DNA (types 16, 18, 31, 33, 35, 45, 51, 52, 58, and 68). Women with abnormal cytological results are referred for colposcopy and biopsies, as indicated according to the Brazilian Ministry of Health Guidelines. HIV history and pertinent laboratory results are obtained from the HIV clinical database [26].

This study included women who enrolled in the Women’s HIV Cohort between 2005 and 2013. From 2005 through 2013, all women entering the cohort underwent cervical linear-array testing for HPV genotypes along with routine Pap smear testing at cohort entry. Cervical samples were collected using a cervical brush or swab and stored in conservative media (STM, Qiagen, Valencia, CA, USA). DNA was extracted by phenol-chloroform standard method. Five μL aliquots were used for HPV detection and genotyping using the Linear-Array HPV Genotyping Test (Roche Molecular Systems Inc., Alameda, CA, USA) which targets low-risk HPV types (6, 11, 26, 40, 42, 53, 54, 55, 61, 62, 64, 66, 67, 69, 70, 71, 72, 73, 81, 82, 83, 84, IS39, and CP6180) and high-risk (oncogenic) HPV types (16, 18, 31, 33, 35, 39, 45, 51, 52, 56, 58, 59, and 68). Women with inadequate specimens or failed genotype testing were excluded from the study. HPV type designation of low-risk or high-risk (oncogenic) was performed in accordance to internationally-recognized classification [27]. Cervical cytology analysis was performed at the INI Pathology Laboratory using standardized methods and the Bethesda rating system: atypical squamous cells unknown significance (ASC-US), atypical glandular cells (AGC), low-grade squamous intraepithelial lesion (LSIL), atypical squamous cells – cannot exclude high-grade lesion (ASC-H), high-grade squamous intraepithelial lesion (HSIL), or cancer. For analyses, AG-US and ASC-H results were included with ASC-US.

We first described the distribution of low- and high-risk HPV types observed among all women. For descriptive purposes, overall prevalence rates and 95 % confidence intervals based upon normal distributions were calculated for the most frequently occurring types. We next examined the prevalence of each high-risk HPV type and of more than one high-risk type by cervical cytology outcome (normal, ASC-US, LSIL, and HSIL or cancer), defined as the proportion of women with the cytological outcome who were positive for the high-risk HPV type. To examine patterns of clustering of high-risk HPV types, we graphically examined the relative frequencies of each high-risk HPV pairing observed in women with abnormal cytology.

We compared patient characteristics at the time of HPV testing associated with HPV genotype subsets: women uninfected with HPV, women infected with only low-risk HPV types, women infected with one high-risk HPV type, and women infected with more than one high-risk HPV type. Demographic and social factors included age, race, education, living situation, income, and tobacco use. We also examined sexual and health behavior, as well as HIV clinical variables including CD4+ lymphocyte nadir (defined as lowest recorded CD4+ lymphocyte prior to and up to 90 days after the HPV testing date), current CD4+ lymphocyte count (closest recorded value within one year), current HIV RNA value (up to one year prior), and total months since initiation of combination antiretroviral therapy (cART). Comparisons of HPV genotype group were performed using Wilcoxon rank sum and Fisher exact tests for continuous and categorical variables, respectively.

To study the association of each high-risk HPV type and presence of more than one high-risk type with abnormal cytology outcomes, we calculated prevalence ratios using logarithmic binomial regression. Prevalence ratios for abnormal cytology (defined as cytology results of ASC-US or higher) by demographic (age, race, education), social (tobacco use, presence of HIV-infected sexual partner), and clinical (CD4+ lymphocyte nadir and count, HIV RNA, and cART history) factors were first examined using unadjusted analyses (data not shown). Unadjusted prevalence ratios of each high-risk HPV type and presence of more than one high-risk HPV type for abnormal cytology were calculated. To develop the most parsimonious model possible, demographic and clinical variables highly statistically significantly associated with abnormal cytology (*p* <0.01) were retained in adjusted models. Final models for each high-risk HPV type included the individual high-risk HPV type, age, CD4+ lymphocyte count, and presence of more than one high-risk HPV. In adjusted analyses, the detection of more than one high-risk HPV type reflects the relative risk of abnormal cytology associated with multiple high-risk HPV types, after accounting for the detection of the individual high-risk type included in the model, age, and CD4+ lymphocyte count. The individual high-risk type included in the model in the adjusted model reflects the risk of abnormal cytology adjusting for background risk associated with co-infections, age, and CD4+ lymphocyte count. To avoid co-linearity, for each model, presence of more than one high-risk type does not by definition include only those co-infections involving individual high-risk HPV of the model. *P* values were adjusted for multiple comparisons by Bonferroni correction in the final adjusted models (statistically significant threshold of *p* < 0.004).

Along with HPV types 26, 66, 67, 70, 73, and 82, HPV 53 has been noted to be possibly oncogenic and may not truly represent low-risk infection [28, 29]. As HPV 53 was found to be highly prevalent in our cohort, we examined its prevalence, clustering, and association with abnormal cytology using the same methods as described for the high-risk HPV types.

Statistical analyses and figures were performed using Stata 12.1 (Stata Corporation, College Station, Texas, USA). All *p* values were two-sided.

## Results

From 2005 to 2013, 590 women enrolled in the women’s cohort and underwent HPV linear array genotyping at the time of cervical cancer screening. HPV genotype results for 28 women (4.7 %) failed or were incomplete, resulting in 562 women who were included in our study. Women with failed genotype testing who were excluded were statistically similar to those included with respect to age, race, education, and sexual history (data not shown). Excluded women had similar CD4+ lymphocyte counts at cohort entry compared to women included in the analyses (median 437 vs. 436 cells/mL respectively, *p* = 0.67). Excluded women had a higher proportion of failed hybrid capture tests (25 vs. 1 %, *p <* 0.001); however, among women with hybrid capture results, excluded and included women had similar rates of positive hybrid capture results (57 vs. 44 % respectively, *p =* 0.27). Lastly, excluded and included women had similar proportions of abnormal cervical cytology outcomes (19 vs. 30 % respectively, *p* = 0.38).

**Fig. 1:**
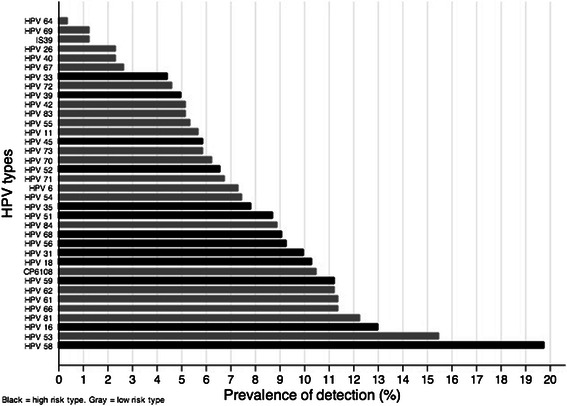
Prevalence of all HPV types. Includes HPV genotype data on all 562 women

The frequencies of each HPV type observed in the cohort are shown in Fig. 1. Overall, the most frequent HPV type observed was high-risk type 58 (prevalence: 19.8 %, 95 % confidence interval [CI]: 16.4–23.1). After HPV 58, HPV 16 was the second most frequently observed high-risk HPV detected (prevalence: 13 %, 95 % CI: 10.2–15.8). HPV 18 was detected in 10.3 % of women (95 % CI: 7.8–12.8). Infection by more than one high-risk HPV type was observed in 32.2 % of women (95 % CI: 28.3–36.1).

Table 1 describes the demographic, social, and clinical characteristics of women included in analyses according to HPV genotype result. Overall, 364 women (65 %) had at least one high risk HPV type detected and nearly half of those women had more than one high risk type present (*n* = 181). Women with infection by more than one high-risk type were younger than women with only one high-risk HPV type detected and were more likely to report a known HIV-infected sexual partner. They did not differ in reported condom use, tobacco, or hormonal contraception use. Women with more than one high-risk type had lower CD4+ lymphocyte count nadirs compared to women with no HPV types detected but did not differ from women with only one high-risk HPV type detected. However, women with more than one high-risk type had lower CD4+ lymphocyte counts at the time of HPV testing compared to both HPV negative women and those with only one high-risk type detected. Women with more than one high-risk type had a greater number of low-risk HPV types detected and were more often infected with HPV 16 or 18. Women with any high-risk type more frequently had abnormal cytology, with presence of more than one high-risk type associated with even higher frequency of abnormal cytology.

**Table 1:** Demographic, social, and clinical characteristics of women by HPV genotype result

	No HPV	Only low risk HPV	One high risk HPV	More than one high risk HPV	Total
N (%)	64 (11.4)	134 (23.8)	183 (32.6)	181 (32.2)	562
Age in years, median (IQR)	37.4 (30.3–41.3)	38.5 (31.1–45.5)	36.0 (29.9–44.0)	32.7 (26.5–41.3)^a^	35.8 (29.3–43.6)
Non-white race, n (%)	50 (79.4)	99 (73.9)	129 (71.3)	124 (68.9)	402 (72.0)
Years of formal education, median (IQR)	8 (5–11)	8 (5–11)	8 (5–11)	9 (6–11)^b^	8 (5–11)
Monthly income (US$), median (IQR)	400 (255–600)	425 (250–750)	400 (232.5–750)	450 (232.5–800)	415 (235–750)
Married/living with partner, n (%)	34 (53.1)	85 (63.4)	100 (54.6)	102 (56.4)	321 (57.1)
Age at sexual debut, median (IQR)	16 (14–18)	16 (15–18)	16 (15–18)	16 (15–18)	16 (15–18)
Number of lifetime sexual partners, median (IQR)	5 (4–12)	5 (3–8)	5 (3–10)	5 (3–9)	5 (3–10)
Condom use with last sexual intercourse, n (%)	37 (57.8)	86 (65.7)	117 (65.4)	118 (66.3)	358 (64.9)
HIV-infected current sexual partner, n (%)	9 (14.1)	38 (28.4)^c^	28 (15.3)	50 (27.6)^c, d^	125 (22.2)
Number of pregnancies, median (IQR)	2 (1–4)	3 (2–4)	3 (1–4)	2 (1–3)^a^	2 (1–4)
Current hormonal contraceptive use, n (%)	16 (25.0)	19 (14.3)	27 (14.8)	33 (18.2)	95 (17.0)
Current tobacco use^e^, n (%)	10 (15.6)	37 (20.6)	36 (20.2)	31 (23.1)	114 (20.5)
Nadir CD4+ lymphocyte count^f^, median (IQR)	302 (190–570)	307 (155–500)	255 (128–483)	256 (90–414)^b^	274 (128–467)
CD4+ lymphocyte count at HPV test^g^, median (IQR)	528 (326–827)	512 (309–679)	433 (309–631)	398 (251–549)^a, b^	436 (296–679)
HIV RNA <400 copies/ml^h^, n (%)	27 (42.2)	63 (47.0)	74 (40.4)	62 (34.3)	226 (40.2)
Total months since cART initiation at HPV test, median (IQR)	1.65 (0–12.5)	2.5 (0–15.1)	3.6 (0–16.9)	2.0 (0–13.5)	2.5 (0–14.13)
Total number of low-risk HPV types detected, median (IQR)	0	1 (1–2)	1 (1–2)	2 (1–3)^a^	1 (0–2)
HPV 16 or 18 detected, n (%)	0	0	42 (23.0)	82 (45.3)^d^	122 (22.1)
Cytology results, n (%)					
Any Abnormal	7 (10.9)	22 (16.4)	53 (24.0)^c^	84 (46.4)^c, d^	166 (29.5)
ASC-US or AGC	5 (7.8)	14 (10.5)	19 (10.4)	26 (14.4)	64 (11.4)
LSIL	1 (1.6)	7 (5.2)	30 (16.4)^c^	47 (26.0)^c, d^	85 (15.1)
HSIL	1 (1.6)	1 (0.8)	4 (2.2)	10 (5.5)	16 (2.9)
Cancer	0	0	0	1 (0.6)	1 (0.2)

^a^Rank sum test of comparison with one HR HPV group *p* < 0.05

^b^Rank sum test of comparison with no HPV group *p* < 0.05

^c^Fisher exact test of comparison with no HPV group *p* < 0.05

^d^Fisher exact test of comparison with one HR HPV group *p* < 0.05

^e^There were 6 women in total missing tobacco use data

^f^There were 5 women in total missing CD4+ lymphocyte nadir data

^g^There were a total of 37 women with missing CD4+ lymphocyte data at the time of HPV exam

^h^There were 63 women with missing HIV RNA data at the time of HPV exam

*Abbreviations used*

*IQR*: interquartile range

*cART*: combination antiretroviral therapy

*ASC-US*: atypical squamous cells of unknown significance

*AGC*: atypical glandular cells

*LSIL*: low-grade squamous intraepithelial lesion

*HSIL*: high-grade squamous intraepithelial lesion

**Fig. 2:**
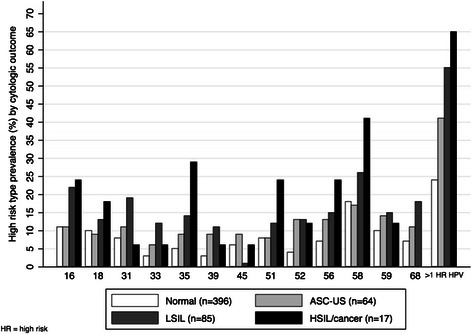
Prevalence of high-risk HPV types and presence of more than one high-risk HPV types by cytology. Abbreviations used: HR: high-risk; ASC-US: atypical squamous cells of unknown significance, includes atypical glandular cells (*n* = 1); LSIL: low-grade squamous intraepithelial lesion; HSIL: high-grade squamous intraepithelial lesion

The prevalence of high-risk HPV types and presence of more than one high-risk type by cytology result are shown in Fig. 2. Overall, HPV 58 was the most common high-risk HPV type detected in women with normal cytology, ASC-US, LSIL, and HSIL or cervical cancer. HPV 16 was detected in 22 % (95 % CI: 13–31 %) of women with LSIL and 24 % (95 % CI: 10–46 %) of women with HSIL or cancer. HPV 18 was detected in 13 % (95 % CI: 6–20 %) and 18 % (95 % CI: 0–38 %) of women with LSIL and HSIL or cancer, respectively. Prevalence of infection by more than one high-risk type increased from 24 % (95 % CI: 20–29 %) among women with normal cytology to 65 % (95 % CI: 36–89 %) among women with HSIL or cancer.

**Fig. 3:**
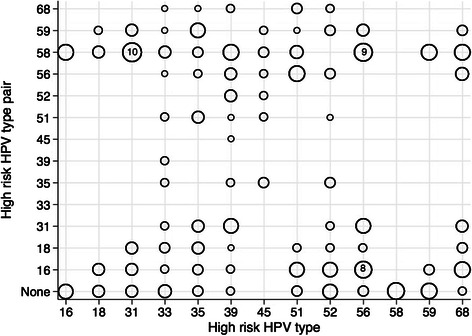
Relative frequencies of high-risk HPV pairings in women with abnormal cytology. Includes results from 166 women with abnormal cervical cytology. Circle size reflects relative frequency of occurrence such that smaller circles reflect less frequent observations. The most frequently observed pairings are labeled (HPV 31 and HPV 58, *n* = 10 occurrences; HPV 56 and HPV 58, *n* = 9 occurrences; HPV 56 and HPV 16, *n* = 8 occurrences). Pair = None refers to HPV type frequency alone, without another high risk HPV type

We next examined the pairing frequencies of high-risk HPV types observed in women with abnormal cytology results (*n* = 166). Figure 3 displays each high-risk HPV pair observed whereby the size of the circle at the intersection of each high risk HPV type corresponds to the relative frequency of the observed pairing. The circles representing the intersection of each high-risk type and “none” reflect the relative frequencies of each high-risk type occurring in monoinfection. Among women with abnormal cytology, HPV 58 was the most common high-risk type detected in monoinfection and in multiple infections. However, the frequencies of individual high-risk pairings were relatively low and distributed across all high-risk HPV types. The pairing combinations of HPV 58 and 31 (*n* = 10, prevalence = 6 %), 58 and 56 (*n* = 9, prevalence = 5 %), and 16 and 56 (*n* = 8, prevalence = 5 %) were the most frequently observed high-risk HPV pairs. HPV 16 paired most often with HPV 58 (*n* = 7) and 68 (*n* = 7). Some high-risk HPV types occurred more frequently in paired infections than monoinfection, including HPV 39 and 68.

Results from unadjusted and adjusted logarithmic binomial regression models for abnormal cytology are reported in Table 2. In unadjusted analyses, a number of individual high-risk HPV types were statistically associated with abnormal cytology, including HPV 16, 31, 33, 35, 39, 52, and 68. In addition, age (prevalence ratio [PR] per 5 year increase 0.92 [95 % CI: 0.86–0.98]), CD4+ lymphocyte count (PR per 100 cells/ml increase 0.90 [95 % CI: 0.85–0.95]), and presence of more than one high risk HPV type (PR 2.16 [95 % CI: 1.68–2.76]) were statistically associated with abnormal cytology results in unadjusted analyses. Notably, tobacco use, number of lifetime sexual partners, condom use, HIV RNA, and cART history were not statistically associated with abnormal cytology in unadjusted analyses (data not shown). In adjusted models adjusting for multiple comparisons, the prevalence ratio point estimates for each high-risk HPV type were attenuated and no longer statistically significant, based upon a Bonferroni corrected *p* value of <0.004 for significance. However, in every adjusted model, presence of more than one HPV type remained strongly associated with an approximate 2-fold increased risk of abnormal cervical cytology, regardless of the individual high-risk HPV type assessed in each model.

**Table 2:** Unadjusted and adjusted prevalence ratios for abnormal cytology of high-risk HPV types

	Unadjusted Prevalence Ratio (PR)		Adjusted Prevalence Ratio (aPR)^a,b^			
High risk HPV type	HPV type PR [95 % CI]	*P* value	HPV type aPR [95 % CI]	*P* value	More than one high risk HPV aPR [95 % CI]	*P* value
16	1.48 [1.08–2.01]	0.013	1.14 [0.83–1.54]	0.418	1.94 [1.50–2.52]	<0.001^c^
18	1.19 [0.81–1.74]	0.369	0.83 [0.57–1.20]	0.321	2.04 [1.57–2.66]	<0.001^c^
31	1.53 [1.09–2.13]	0.013	0.97 [0.70–1.37]	0.882	1.98 [1.51–2.59]	<0.001^c^
33	2.13 [1.51–3.02]	<0.001	1.47 [1.10–1.97]	0.009	1.89 [1.45–2.46]	<0.001^c^
35	1.89 [1.38–2.59]	<0.001	1.20 [0.88–1.64]	0.246	1.88 [1.43–2.48]	<0.001^c^
39	2.03 [1.43–2.88]	<0.001	1.09 [0.74–1.61]	0.665	1.95 [1.49–2.53]	<0.001^c^
45	0.80 [0.44–1.50]	0.507	0.58 [0.32–1.06]	0.079	2.07 [1.60–2.68]	<0.001^c^
51	1.35 [0.93–1.97]	0.116	1.00 [0.70–1.43]	0.993	1.97 [1.52–2.56]	<0.001^c^
52	2.05 [1.50–2.81]	<0.001	1.35 [1.00–1.84]	0.051	1.89 [1.45–2.46]	<0.001^c^
56	1.74 [1.27–2.38]	0.001	1.06 [0.76–1.47]	0.726	1.94 [1.48–2.55]	<0.001^c^
58	1.29 [0.97–1.72]	0.084	0.90 [0.68–1.21]	0.488	2.04 [1.56–2.68]	<0.001^c^
59	1.34 [0.95–1.89]	0.097	1.00 [0.72–1.38]	0.976	1.97 [1.51–2.57]	<0.001^c^
68	1.53 [1.09–2.16]	0.015	1.04 [0.74–1.46]	0.834	1.96 [1.50–2.56]	<0.001^c^

^a^Due to missing CD4+ lymphocyte values in 37 women, 525 women were included in multivariate models

^b^All adjusted models included individual high-risk HPV type, age, CD4+ lymphocyte count at HPV test, and presence of more than one high risk HPV types. CD4+ lymphocyte count and presence of more than one high-risk HPV types remained statistically significant. Age was not statistically significant in multivariate models

^c^*p* value significant at Bonferroni threshold (0.004) for correction of multiple comparisons

*Abbreviations used*

*CI*: confidence interval

*PR*: prevalence ratio

*aPR*: adjusted prevalence ratio

The second most frequently observed type was HPV 53 (prevalence: 15.5 %, 95 % CI: 12.5–18.5 %) and analyses examined its epidemiology given its weakly carcinogenic risk. Of the 87 women infected with HPV 53, 67 (77 %) were also infected with a high-risk HPV type. Thirty-two (19 %) of all women with HPV 53 infection had abnormal cytology; however, 31 of these women were also infected with high-risk HPV types. One woman with HPV 53 infection without co-infection by a high-risk type was found to have ASC-US on cytology. In unadjusted analyses, infection by HPV 53 was associated with increased risk of abnormal cytology (PR = 1.3, 95 % CI: 0.96–1.78). In a model adjusted for age, CD4 lymphocyte count, and co-infection with high-risk types, the association was no longer observed (aPR = 0.90, 95 % CI 0.67–1.21).

## Discussion

In this large study of HIV-infected women, we found that infection with high-risk HPV types and infection with multiple high-risk types, in particular, were highly prevalent and associated with abnormal cervical cytology. While HPV 16 infection was common, HPV 58 was the most common HPV type detected overall. Our study highlighted the role of infection by more than one high-risk type. Individual pairing frequencies of high-risk HPV types were relatively low overall; however, the presence of multiple high-risk HPV types was strongly associated with abnormal cytology outcomes, even when adjusting for detection of individual high-risk HPV types.

In this cohort, infection by high-risk HPV types was common, occurring in more than half of all women. Infection with high-risk HPV types was associated with younger age and lower CD4+ lymphocyte counts. These findings are consistent with previous studies of this cohort and other studies of HIV-infected women in Brazil [15, 30–33]. Differing from other studies, tobacco use was not associated with high-risk HPV infection or abnormal cervical cytology [32, 34]. Previous work examining risk of high-grade cervical intra-epithelial neoplasia by histopathology among women at our clinic demonstrated an association with tobacco use [35]. The lack of association observed in this analysis may be a result of differing outcome measures.

Additionally, infection with more than one high-risk HPV type was highly prevalent. Women with infections by more than one high-risk type were notably younger and had lower CD4 lymphocyte counts compared to women with high-risk HPV monoinfection. Increased risk of infection by multiple high-risk HPV types has been associated in HIV-infection and younger age [36–39]. We also observed higher frequency of known HIV-infected sexual partners among women with multiple high-risk HPV infection compared to those with only one high-risk HPV detected. While the HIV infection status of the partner may have affected sexual practices (though no difference in condom use was reported between the groups), studies of HIV serodiscordant couples have shown HIV infection increases the risk of acquiring HPV infection from uninfected partners in both women and men [40]. The HIV infection status of sexual partners thus may be an important risk factor for HPV infection among HIV-infected women.

In our study, presence of more than one high-risk type was consistently and independently associated with risk of abnormal cytology, after adjusting for presence of individual high-risk types (including HPV 16), age, and CD4+ lymphocyte count. Infection by more than one high-risk HPV type has been associated with increasing risk of cervical intraepithelial neoplasia [38, 41]. A recent study of >59,000 cervical cytology specimens and HPV genotypes from New Mexico demonstrated that, with the exception of HPV 16, HPV infection by more than one high-risk type conferred additional risk of HSIL compared to monoinfection for each high-risk type examined. However, risk was not further increased when more than two high-risk types were detected, suggesting that risk of high-grade cervical outcomes is not synergistically increased with multiple infections [41]. While clustering of high-risk HPV types is common, as observed in our study, patterns of specific pairings tend to be unpredictable. Studies that have statistically examined clustering patterns of high-risk HPV types have generally concluded that specific high risk HPV pairings occur at random [42–44]. Nonetheless, the limited sample size of most studies precludes statistical inference regarding patterns of clustering and their impact on the natural history of cervical cancer. This highlights the importance of international collaborative studies and meta-analysis in HIV and HPV research [45].

In this study, high-risk HPV 58 was the most common HPV type detected in all women, including those with multiple high-risk types detected and those associated with abnormal cytology. Worldwide, HPV 16 is the most frequently detected high-risk type, including in HIV-infected women [13]. However, the prevalence of high-risk types other than 16 and 18 is higher in HIV-infected women compared to uninfected women [13, 14]. HPV 58 has been observed at high rates in multiple studies of cervical infection in women in Brazil and has been observed at rates above those of HPV 16 in cohorts of HIV-infected women in Botswana and Zambia [32, 46–49]. Globally, HPV 58 is the third most prevalent HPV type detected in cases of cervical cancer [50]. While HPV 58 was the most frequent high-risk HPV type observed in women with abnormal cytology, further study is needed in its association with histological measures of neoplasia, particularly in HIV-infected women. In a longitudinal study of HIV-uninfected women, HPV 58 was not as strongly associated with cervical intraepithelial neoplasia as HPV 16 or 31 [51]. However, the pathogenicity of HPV 58 in HIV-infected women has not been described.

Understanding of epidemiology of specific HPV types, particularly in high-risk populations like HIV-infected women, is of critical importance for HPV vaccine development and implementation. While studies have suggested cost-effectiveness, general access to the HPV vaccines is not currently available in Brazil [25]. Importantly, second-generation HPV vaccines will include coverage for a greater number of high-risk HPV types, including 6,11,16,18, 31, 33, 45, 52, and 58, which could be particularly important in our population where HPV 58 is highly prevalent. Mathematical models of second-generation HPV vaccines have predicted even greater absolute risk reduction of cervical cancer compared to current vaccines. However, models have suggested that these effects may be blunted by infection by more than one high-risk type [52]. This study highlights the need for HPV vaccines that cover for a diversity of high-risk HPV types and also the need for further information about their effectiveness in settings of multiple infection.

Our study has important strengths and limitations. First, this study was designed as a cross-sectional analysis, which limits our ability to draw conclusions regarding causality. Additionally, without longitudinal genotype data available, we did not examine HPV infection clearance or long-term cytological and histological outcomes in this analysis. An important strength of our study is the large cohort of women included for which detailed health and behavioral information was available through questionnaires. This study reflects the largest epidemiologic study of HPV genotypes in HIV-infected women in Latin America. Nonetheless, women who chose to participate in the cohort may differ with those who declined and, given the single-center catchment, results from our study may not be generalizable to other settings. It is notable to highlight that all women who entered the cohort during the period of 2005-2013 underwent HPV genotype testing, thus minimizing the risk of selection bias of those tested. We used any abnormal cytology results as a composite outcome of interest rather than LSIL or HSIL. By including ASC-US outcomes, which are not as strongly associated with cervical intraepithelial neoplasia, we may have diminished the ability to detect risk conferred by specific high-risk HPV types and may have underestimated the effects of infection by more than one high-risk HPV.

Finally, we included in our analyses those high-risk types most strongly considered oncogenic. Recently, a number of HPV types previously considered low-risk have been recognized to have some oncogenic potential, namely HPV 26, 53, 66, 67, 69, 70, 73, and 82 [28, 29, 53]. Detection of these viruses would not have occurred using hybrid capture alone and our study expands the available data about their epidemiology. HPV 53 was the second most common HPV type detected overall and was associated with abnormal cytology in unadjusted analyses. However, only one woman with monoinfection by HPV 53 had abnormal cytology (ASC-US) and the association of HPV 53 with abnormal cytology was markedly attenuated after adjusting for presence of infection by more than one high-risk type. Further study into the persistence, clearance, and long-term outcomes associated with these possibly oncogenic HPV types is needed.

## Conclusions

In conclusion, in a large study of HPV epidemiology in a cohort of HIV-infected women in Brazil, we found that infection by high-risk HPV types and infection by more than one high-risk type were highly prevalent. Infection by more than one high-risk type was strongly associated with abnormal cytology, even after adjusting for age, CD4+ lymphocyte count, and specific high-risk HPV types. HPV type 58 was the most common HPV type detected overall and among abnormal cytological outcomes. Further studies on its risk for cervical intraepithelial neoplasia and longitudinal outcomes are needed. The diversity and frequency of infections by more than one high-risk HPV may have important implications on the effectiveness of second-generation HPV vaccines. However, the prevalence of high-risk HPV infections in this high-risk population underscores the importance of all methods of cervical cancer prevention, including regular screening and vaccine availability.
